# Validity and reproducibility of the Physical Activity Scale for the Elderly (PASE) questionnaire for the measurement of the physical activity level in patients after total knee arthroplasty

**DOI:** 10.1186/1471-2474-15-46

**Published:** 2014-02-20

**Authors:** Sylvain Bolszak, Nicola C Casartelli, Franco M Impellizzeri, Nicola A Maffiuletti

**Affiliations:** 1 Institute of Human Movement Sciences and Sport, ETH Zurich, Zurich, Switzerland; 2 Neuromuscular Research Laboratory, Schulthess Clinic, Zurich, Switzerland; 3 INSERM U1093 - Cognition, Action and Sensory Plasticity, University of Burgundy, Dijon, France

**Keywords:** Physical Activity, Questionnaire, Arthroplasty, Knee

## Abstract

**Background:**

The need for valid and reproducible questionnaires to routinely assess the physical activity level of patients after total knee arthroplasty (TKA) is of particular concern in clinical settings. Aims of this study were to evaluate the validity and reproducibility of the physical activity scale for the elderly (PASE) questionnaire in TKA patients, with a particular view on gender differences.

**Methods:**

A total of 50 elderly patients (25 women and 25 men aged 70 ± 6 years) following primary unilateral TKA were recruited. The reproducibility was evaluated by administering the PASE questionnaire during two occasions separated by 7 days. The construct (criterion) validity was investigated by comparing the physical activity level reported by patients in the PASE questionnaire to that measured by accelerometry. Reproducibility was evaluated using intraclass correlation coefficients (ICC_3,1_) for reliability and standard error of measurement (SEM) and smallest detectable change (SDC) for agreement, while validity was investigated with Pearson correlation coefficients.

**Results:**

Reliability of the PASE total score was acceptable for men (ICC = 0.77) but not for women (ICC = 0.58). Its agreement was low for both men and women, as witnessed by high SEM (32% and 35%, respectively) and SDC (89% and 97%, respectively). Construct validity of the PASE total score was low in both men (*r* = 0.45) and women (*r* = 0.06).

**Conclusions:**

The PASE questionnaire has several validity and reproducibility shortcomings, therefore its use is not recommended for the assessment of physical activity level in patients after TKA, particularly in women.

## Background

Knee osteoarthritis is a frequently occurring debilitating disease which may cause joint pain, lower extremity muscle weakness and physical dysfunction in elderly men and women [1]. At the present time, the most effective surgical treatment for end-stage knee osteoarthritis is total knee arthroplasty (TKA) as it is successful in relieving pain and improving functional ability in approximately 80% of patients [1-4]. The number of TKA procedures performed in developed countries has increased by over 25% in the last decade, partly due to population aging [2,3], with about two thirds of all TKAs performed on women [5]. As knee replacement reduces knee pain and improves physical function [2,6], this should allow patients to increase their physical activity level (PAL). However, although TKA patients report improvements in their PAL 12 months after surgery (compared to preoperative), they still do not reach the recommended amount of physical activity [7]. On the other hand, high levels of sport and heavy labor activities have been mentioned as important risk factors for early implant failure [8]. Thus, assessment of PAL using valid and reproducible measurement tools is of particular concern for patients after TKA [9].

The doubly-labeled water method is considered to be the gold standard for measuring physical activity [10]. However, its implementation is quite complex, has considerable costs and is therefore unrealistic in a clinical setting. A more popular tool to assess the PAL is portable accelerometry, which consists of recording a person’s body motion in free-living conditions [11]. Accelerometric systems are able to provide the duration and the intensity of physical activities, as well as the associated energy expenditure. Therefore, they are often used to validate physical activity questionnaires because they measure closely-related constructs [12]. Nevertheless, the practicability of accelerometers in routine assessment of physical activity is not usual because of their cost and the need of a technician for data handling. Recall questionnaires are commonly used in large-scale trials and epidemiological studies since they are inexpensive, easy to administer, and thus more appropriate than accelerometers to estimate PAL in a clinical setting [11]. However, up to now, no questionnaire has proven to be valid and reproducible for the assessment of the PAL in patients after TKA [13].

The physical activity scale for the elderly (PASE) is a recall questionnaire that was specifically developed for people aged 65 years and older. It assesses intensity, frequency and duration of physical activities and can be self- or interviewer-administered. The advantages of the PASE questionnaire are that it takes a short time to be completed, the recall time frame is short and it especially considers low-intensity activities (light leisure and household activities) which are commonly performed by the elderly [14]. Additionally, the PASE questionnaire has recently been recognized as a promising tool for the assessment of physical activity in knee osteoarthritis patients [13]. The PASE total score has been found to be moderately correlated with direct methods for assessing PAL (doubly-labeled water and accelerometry), but also with grip strength, static balance, knee muscle strength and 6-min walking distance in patients with knee osteoarthritis and in healthy subjects [14-21]. However, to date, the validity and reproducibility of the PASE questionnaire for the assessment of physical activity in patients with TKA has never been examined.

The main aims of this study were to evaluate the validity (using accelerometer measures as criterion) and reproducibility of the PASE questionnaire in TKA patients. A special focus was devoted to sex-related differences because some of the above-cited validation studies reported better correlations between PASE total score and objectively measured PAL in men than in women [16,17,19].

## Methods

### Subjects

Because a sample size ≥ 50 should be used to evaluate the validity and reproducibility of physical activity questionnaires [12], a total of 50 consecutive TKA patients (25 men and 25 women) were included in the study (Table 1). The main inclusion criterion was primary unilateral TKA implanted at the Schulthess Clinic (Zurich, Switzerland) from April 2011 to March 2012. Specifically, patients were evaluated between 3 and 12 months postoperatively, because physical function and pain have recently been shown to attain preoperative values 3 months after TKA surgery, but no further significant PAL improvements have been reported 12 months postoperatively [2,7]. Exclusion criteria were other artificial joints in the lower extremities, and symptoms or signs referable to overt cardiorespiratory, orthopedic, neurological or general diseases that could have negatively influenced the physical activity evaluation with questionnaires or accelerometers. The study was conducted according to the Helsinki declaration, and the protocol was approved by the Ethics Committee of the Canton of Zurich. All the subjects signed a written informed consent form before participating in the study.

**Table 1:** Characteristics and sociodemographic data of the patients

	**Men**	**Women**
*n*	25	25
Age (yrs)	68.9 ± 5.2	70.0 ± 6.1
BMI (kg∙m^-2^)	28.0 ± 3.8	25.7 ± 3.5*
Time post operation (month)	8.3 ± 2.9	7.9 ± 2.7
WOMAC total score	95.3 ± 4.6	94.9 ± 3.8
WOMAC pain	96.3 ± 4.2	95.9 ± 4.4
WOMAC stiffness	92.0 ± 6.8	92.6 ± 6.3
WOMAC function	95.4 ± 5.5	94.9 ± 4.1
Living situation (%)		
Urban	60	32
Rural	40	68
Occupation (%)		
Retired	72	84
Part time job	12	4
Full time job	16	12

BMI, Body Mass Index. WOMAC, Western Ontario and McMaster Universities Arthritis Index. Significant difference between men and women at level *: P < 0.05.

### Study design and experimental procedures

Patients were invited to attend the laboratory on two occasions, separated by 7 days. They were explicitly asked to maintain their usual physical activity habits between the two test sessions. Therefore, we assumed that all patients maintained their usual PAL during this time period. During the first test session (hereafter referred to as test session 1), patients were initially asked to fill in a questionnaire enquiring about their sociodemographic characteristics, and the Western Ontario and McMaster Universities Arthritis Index (WOMAC) questionnaire. The WOMAC is a valid and reliable questionnaire widely used for evaluating knee pain, stiffness, and function in patients with osteoarthritis of the knee [22]. It has 24 items, and the scores vary between 0 (worst score) and 100 (best score). Patients were then asked to complete the PASE questionnaire. Subsequently, they were instructed to wear a portable accelerometer as much as possible until test session 2. The accelerometer was attached on an elastic belt and firmly fixed on the right hip. Patients were advised to remove the accelerometer for sleeping and during water-related activities (e.g., showering, swimming, water-gymnastic), and to note the wearing and non-wearing time periods in a daily log. Patients brought the accelerometer back at test session 2, and filled out the PASE questionnaire again. All patients received the same instructions by the same interviewer, and filled in the questionnaire together with the interviewer. Test-retest reproducibility was investigated by comparing the PASE scores obtained at test session 1 and 2. The construct validity of the PASE questionnaire was investigated by comparing the PASE scores at test session 2 to the physical activity outcomes measured by accelerometry.

### PASE questionnaire

The PASE is a 12-item scale evaluating the PAL of the past 7 days in three life domains: recreational, household and work-related activities. In the present study, the PASE was administered through an interviewer, because this modality has proven to be more reliable than the self-administered one [14]. Patients were asked to rate the weekly frequency and daily duration for each of the following recreational activities: walking outside the home, light, moderate and strenuous activities and muscle strengthening. Whether household activities (light and heavy housework, home repairs, lawn work/yard care, outdoor gardening and caring for others) were performed was captured by answering yes or no. Finally, working for pay or as a volunteer was assessed by recording the amount of hours per week and the type of work performed. For each activity a score was obtained by multiplying an activity frequency value by a task-specific weight provided by the scoring manual. The main outcomes are PASE total score and activity and intensity PASE sub-scores. The PASE total score, which represents the overall PAL, is the sum of all activities together, and ranges between 0 and 400 or more. The activity sub-score for recreational, household and work-related activities are computed by adding the activities corresponding to each life domain together [14]. Additionally, with an explorative intention, we calculated two PASE intensity sub-scores: one for low intensity activities and one for moderate-to-high intensity activities. We first determined, according to the physical activity compendium [23], the average metabolic equivalents of task (MET) for each item of the PASE. The classification of each single PASE questionnaire item into the different PASE intensity sub-scores was performed by consensus of two experienced investigators. Subsequently, according to the age-related classification of physical activity intensity provided by the U.S. Department of Health and Human Services [24], we defined the threshold between low and moderate-to-high intensity activities to be 3.5 MET and 4.5 MET for patients aged over and under 65, respectively. For patients older than 65 years (*n* = 40), the sum of the following items represented the low intensity activity PASE sub-score: walking outside the home, light recreational activity, light and heavy housework and caring for others. If the type of work was mainly sitting, or sitting and standing with some walking, the work-related activity score was added to the low intensity activities. Otherwise, it was added to the moderate-to-high intensity PASE sub-score. The sum of the remaining items (moderate and strenuous recreational activities, muscle strengthening, home repairs, lawn work/yard care, outdoor gardening) represented the moderate-to-high PASE sub-score. For patients younger than 65 years (*n* = 10), the score of the items home repairs and outdoor gardening were also included as low intensity activities.

We performed a cross-cultural adaptation of the PASE questionnaire into German according to the guidelines of the American Association of Orthopedic Surgeons Outcomes Committee [25]. The questionnaire was translated from English into German by an informed and an uninformed translator, who were both native German speakers. Then the two versions were combined into one under the supervision of a research methodologist. Two native English speakers, who were fluent in German, performed each a back translation. All involved persons resolved remaining problems and approved the final version of the German PASE questionnaire. Thus, physical activity examples reported in the single PASE items were strictly culturally adapted in order to be familiar to the elderly people living in Switzerland. Finally, the German version of the PASE questionnaire was pre-tested in 15 consecutive patients after total knee or hip arthroplasty to examine comprehensibility of wording and items. The final version was then approved by the New England Research Institute (the copyright owners of the PASE questionnaire).

### Accelerometry

Physical activity was objectively monitored using the Actigraph GT3X + accelerometer (ActiGraph, LLC, Pensacola, FL, USA). It is small (4.6 × 3.3 × 1.9 cm), light-weight (19 g) and measures accelerations in magnitudes of ± 6 g. The accelerometry-derived PAL (aPAL) is characterized by the total number of counts, which is the vector resulting from accelerations in the three orthogonal axes (vertical, antero-posterior and medio-lateral). In the present study, acceleration data was sampled at 100 Hz, filtered, digitalized and added over a user-specified time interval (epoch) of 60 s. Accelerometer data were analyzed and computed with ActiLife 5 software (ActiGraph software, LLC, Pensacola, FL, USA). For each patient, a complete data set was defined to have at least 10 h∙day^-1^ of monitored wear during at least 5 days [26,27]. Nonwear periods were defined as time intervals of at least 60 consecutive minutes of zero counts, with an activity interruption allowance of 0-100 counts∙min^-1^ lasting a maximum of 2 consecutive minutes [28]. The aPAL was calculated by dividing the total amount of counts by the total duration of monitored activity. The time spent per day in low intensity activities and the time spent in moderate-to-high intensity activities were calculated using the equation of Sasaki *et al.*[29] which allows converting Actigraph GT3X + counts into MET (MET = 0.000863∙counts + 0.668876). The same intensity thresholds as those adopted for PASE intensity sub-scores were used. For patients older than 65 years, the threshold between low and moderate-to-high intensity activity was set at 3280 counts∙min^-1^. For patients younger than 65 years, the threshold was fixed at 4323 counts∙min^-1^. Epochs with less than 150 counts∙min^-1^ were classified as inactive and therefore excluded from the analyses [30].

### Statistical analysis

Data normality was evaluated with Shapiro-Wilk tests. Descriptive data are presented as means ± standard deviations (SD), while validity and reproducibility results are presented with the corresponding 95% confidence intervals (CI). Differences between men and women were investigated with unpaired *t* tests (two-tailed). Reproducibility of the PASE total score was investigated using two-way mixed, single measure intraclass correlation coefficients (ICC_3,1_) for reliability, percent standard error of measurements (SEM) and smallest detectable changes (SDC = 1.96 × √2 × SEM) for agreement [31]. Reliability refers to the ability of a measurement instrument to differentiate among patients (despite measurement error), while agreement refers to the precision of the measurement instrument [31]. Acceptable reliability was set at ICC ≥ 0.70 for a sample size of *n* ≥ 50 [12]. In addition, differences between the first and second test sessions were calculated for PASE total score, activity and intensity sub-scores using paired *t*-tests (two tailed). The construct (criterion) validity of the PASE questionnaire was evaluated using Pearson’s correlation coefficients between PASE total score and aPAL. Low and moderate-to-high intensity activity PASE sub-scores were correlated to the min spent in low and moderate-to-high intensity activities, respectively. Adequate validity was set at *r* ≥ 0.50 for a sample size of *n* ≥ 50, according to Terwee *et al.*[12] for examining associations between physical activity questionnaires and accelerometry. Statistical analyses were conducted with the SPSS version 20 (SPSS, Chicago, IL, USA). The significance level was set at *P* < 0.05.

## Results

Descriptive data of the PASE scores are presented in Table 2. Men had a higher work-related activity PASE sub-score at test session 1 (*P* < 0.01) and higher moderate-to-high intensity activity PASE sub-scores at both sessions compared to women (*P* = 0.01 and *P* = 0.02, respectively). Women demonstrated a lower recreational activity PASE sub-score at test session 2 compared to session 1 (*P* = 0.04).

**Table 2:** PASE descriptive data

	**PASE scores**			
	**Test session 1**		**Test session 2**	
	**Mean ± SD**	**%**	**Mean ± SD**	**%**
**Men (*n *** **= 25)**				
Total score	137.1 ± 59.8	100	125.0 ± 58.9	100
Activity sub-score				
Recreational activity	40.8 ± 28.9	30	35.7 ± 25.8	29
Household activity	73.6 ± 40.9	54	70.0 ± 36.2	56
Work-related activity	22.7 ± 42.5**	17	19.3 ± 43.7	15
Intensity sub-score				
Low intensity activity	82.4 ± 43.9	60	75.6 ± 31.2	61
Moderate-to-high intensity activity	54.7 ± 41.3*	40	49.3 ± 46.1*	39
**Women (*n *** **= 25)**				
Total score	116.6 ± 41.2	100	112.1 ± 52.9	100
Activity sub-score				
Recreational activity	49.0 ± 37.1	42	35.9 ± 23.1	32
Household activity	67.6 ± 32.0	58	67.6 ± 32.1	60
Work-related activity	0.0 ± 0.0**	0	8.6 ± 36.1	8
Intensity sub-score				
Low intensity activity	87.7 ± 30.3	75	86.6 ± 47.8	77
Moderate-to-high intensity activity	28.9 ± 29.4*	25	25.5 ± 27.9*	23

PASE, Physical Activity Scale for the Elderly.

Significant difference between men and women at level *: *P* < 0.05; **: *P* < 0.01.

Significant difference between test session 1 and test session 2 at level ^†^: *P* < 0.05.

Two patients (1 woman) were excluded from the reproducibility analyses because they were on holiday the week preceding the study, and this significantly altered their physical activity habits. No significant differences were observed for the PASE total score between the two test sessions, in both men [-10.2% (-23.8 to 47.1)] and women [-9.7% (-24.5 to 8.1)]. ICCs were 0.77 (0.53 to 0.89) and 0.58 (0.25 to 0.80) for men and women, respectively. SEM were 32% (24 to 47%) and 35% (26 to 52%) for men and women, respectively. SDC were 89% (67 to 130%) and 97% (72 to 144%) for men and women, respectively.

On average, patients wore the accelerometer for a total of 6 ± 1 days. There was no wearing time difference between men and women (840 ± 94 min/day and 835 ± 78 min/day, respectively, *P* = 0.40). The aPAL was significantly higher in women than in men (*P* < 0.01), with women spending more time in low intensity activities (*P* = 0.04) (Table 3).

**Table 3:** Accelerometry descriptive data

	**Men (*n*** **= 25)**	**Women (*n*** **= 25)**
	**Mean ± SD**	**Mean ± SD**
aPAL (counts∙min^-1^)	672 ± 215	840 ± 249**
Activity intensity		
Low (min∙day^-1^)	422 ± 114	484 ± 97*
Moderate-to-high (min∙day^-1^)	25 ± 26	29 ± 27

aPAL, Accelerometry derived Physical Activity Level.

Significant difference between men and women at level *: *P* < 0.05; **: *P* < 0.01.

**Figure 1:**
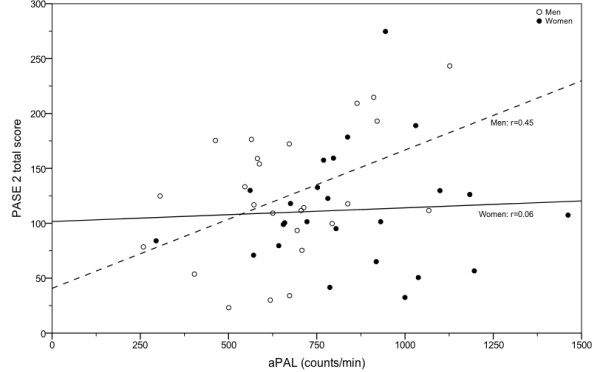
**aPAL vs. PASE 2 total score for men (n = 25) and women (n = 25).** aPAL, Accelerometry derived Physical Activity Level; PASE 2, Physical Activity Scale for the Elderly at test session 2.

Validity results are shown in Table 4 and Figure 1. A moderate and significant correlation was found between the PASE total score and aPAL for men, but not for women. Low and non-significant correlations were found between low and moderate-to-high intensity activity PASE sub-scores and accelerometry-derived minutes spent in low and moderate-to-high intensity activities, respectively.

**Table 4:** PASE construct validity results

	**Men (*n*** **= 25)**		**Women (*n*** **= 25)**	
	***r***	***p*-value**	***r***	***p*-value**
	**Mean (95% CI)**		**Mean (95% CI)**	
PASE total score vs.:				
aPAL (counts∙min^-1^)	0.45 (0.09 to 0.71)	*0.01*	0.06 (-0.24 to 0.36)	*0.39*
PASE sub-scores vs. activity intensity:				
low (min∙day^-1^)	0.22 (-0.13 to 0.53)	*0.15*	-0.01 (-0.30 to 0.30)	*0.48*
moderate-to-high (min∙day^-1^)	-0.14 (-0.41 to 0.30)	*0.25*	0.09 (-0.29 to 0.48)	*0.33*

PASE, Physical Activity Scale for the Elderly; aPAL, Accelerometry derived Physical Activity Level.

## Discussion

The PASE total score demonstrated acceptable reliability for men but not for women and low agreement for both men and women. When compared to accelerometry measures, PASE total score had inadequate validity. The total amount of physical activity, as assessed with accelerometry, was greater in women than in men, but this was not confirmed by the PASE total scores.

There was no evidence of gender-related differences for the PASE total score, but men tended to have higher scores than women, mainly because of higher work-related activity scores. In fact, more men than women in our group of patients were still working part- or full-time. The PASE total scores of our TKA patients were comparable to those previously obtained in healthy populations [15,16,21] and in patients with knee pain and disability [18]. The relatively high PASE scores we observed could be explained, at least in part, by the fact that most TKA patients begin a formal exercise prescription program after surgery and usually maintain it afterwards. In contrast, Tsonga *et al.*[32] reported a mean PASE score of 68 in Greek women 6 months after TKA. These scores are much lower compared to our patients, likely because the group of women evaluated in this previous study was older (73 years), had a greater BMI (30 kg∙m^-2^) and was recruited early after surgery compared to the patients considered in our study.

No systematic bias was found for the PASE total score between the two test sessions, despite a substantial reduction of approximately 10% from test to retest. Personality traits, social desirability and social approval were recognized to be possible sources of systematic bias [33]. Since TKA patients are encouraged by doctors and therapists to exercise, we suppose that patients in this study tended to overestimate their physical activity to attain social approval and desirability of the investigator at the first session [34]. In contrast, knowing that the PASE total score referred to the week objectively assessed by the accelerometer, patients committed to report their physical activities more precisely and truthfully at the second session. This was particularly reflected in the recreational activity sub-score which is dependent on the reported weekly frequency and daily duration, and this resulted in a significantly lower recreational activity sub-score for women.

The PASE total score showed acceptable reliability for men but not for women according to the predefined threshold proposed by Terwee *et al.*[12]. These findings are comparable to those obtained in previous studies investigating the reliability of PASE questionnaire in healthy and pathological elderly populations [14-16,20] The high mean ICC of 0.91 reported by Dinger *et al.*[15] in a rural elderly community can be explained by a substantial difference in the study design compared to the present investigation. In fact, questionnaire administrations were separated by only 3 days and referred to the same week in the study of Dinger *et al.*[15], while in the current study they referred to two consecutive but different weeks. These reliability results indicate an acceptable but moderate ability of the PASE questionnaire to discriminate between male patients following TKA according to their PAL, but not between female patients.

The PASE total score demonstrated low agreement for both men and women due to the large measurement error, with SEM values similar to those recently reported in knee osteoarthritis patients [20]. The smallest detectable change represents the smallest change in the questionnaire score that can be interpreted as a “real change” above measurement error [31]. In order to detect real changes in the questionnaire score after an intervention or over time, the smallest detectable change should be smaller than the minimal clinically important change [12]. Unfortunately, the minimal PASE score change that can be considered clinically relevant in TKA patients is not known. However, the acceptability of PASE agreement results can be interpreted using the noise-to-signal ratio, which can be expressed as an effect size (change in score divided by SEM) [35]. Tsonga *et al.*[32] observed an increase of 18% in the PASE total score of female patients between 3 and 6 months after TKA, while an average PASE total score SEM of 34% was observed in the present study. Interpreting the signal as the change in score (18%), the noise we obtained (34%) was almost 2 times larger than the signal. Since the noise has to be smaller than the changes in score, this suggests that the PASE questionnaire has a very limited ability to distinguish measurement error from real changes. Hence, the PASE questionnaire is not suitable for longitudinal monitoring of the PAL in TKA patients of both sexes.

The PASE total score showed inadequate validity in both men and women. Indeed, although the association between the PASE total score and aPAL was significant for men, the correlation coefficients did not reach the defined threshold of *r* ≥ 0.50 proposed by Terwee *et al.*[12]. In previous studies, the correlations between the PASE total score and aPAL ranged between 0.43 and 0.52 in healthy elderly people [15,17,21] and was 0.30 in patients with hip osteoarthritis [20]. There is no consensus on how high correlations should be to demonstrate adequate validity [36]. Previous studies rated the PASE questionnaire to be valid with significant but lower correlations with respect to those obtained in the present study [14-16,18,20]. We chose to use the more conservative thresholds because the worse the measurement properties are, the higher the risk is for misclassification and biased results [20].

In line with our findings, some studies reported better validity of the PASE total score for healthy elderly men compared to their female counterparts [16,17,19]. This was probably associated to household-related activities, which are generally more frequently performed by women than men [16,19]. However, such an assumption is not supported by our observations as household activity sub-scores did not differ significantly between men and women. We nevertheless acknowledge that household activities might be a potential source of misjudgment for PASE questionnaire scoring, since, contrary to recreational and work-related activities, household activities provide a fixed score without considering frequency and duration.

The PASE total score is the standard outcome for this questionnaire. However, for explorative purposes, we determined two PASE intensity sub-scores. Indeed, differentiating patients by the time spent in low and moderate-to-high intensity activities may be important for investigations focusing on the dose–response effects of physical activity. However, unfortunately, we found no significant correlations between the time spent in low or moderate-to-high intensity activities and the respective PASE sub-scores. Therefore, our results, together with those of Svege *et al.*[20], seem to confirm that the PASE has no ability to differentiate physical activity intensities.

The PASE total score failed to detect the gender-related differences in the total amount of physical activity reported by the accelerometer (women > men). Questionnaires have several limitations associated with recall and reporting bias; they overestimate the time spent on strenuous activities and underestimate activities lasting less than 10 min or with a level of exertion lower than brisk walking [37]. Therefore, we suppose that the mismatch between accelerometry and PASE total score lies in the ability of the PASE questionnaire to assess low intensity activities, since the reported higher aPAL of women is explained by the fact that they spent significantly more time on low intensity activities than men. Therefore, the lack of gender differences in the PASE total score further weaken its construct validity.

The main limitation of this study is the design used to evaluate the reproducibility of the PASE questionnaire. Since the physical activity level was assessed for reproducibility during two consecutive but different weeks, it cannot be ensured that TKA patients maintained the same physical activity level during this time period. The repeated assessment of the physical activity level referring to the same week would have been methodologically more appropriate. However, a time period of less than a week between two questionnaire administrations would have increased the risk of recall bias [38]. In addition, accelerometer wearing could have influenced the reproducibility results, by inducing the TKA patients to be more active during the second week. On the other side, we explicitly asked the patients to maintain their usual physical activity habits during this time period. Two patients demonstrated disparate physical activity habits between the two weeks because of holidays and were thus excluded from reproducibility analyses thereby reducing the statistical power.

## Conclusions

The PASE questionnaire demonstrated acceptable reliability for men but not for women, and low agreement and inadequate validity for both men and women. Accordingly, our findings suggest that there are several shortcomings associated with the use of the PASE questionnaire to assess PAL in TKA patients, particularly in women. Therefore, we do not recommend the use of the PASE questionnaire for evaluating PAL in TKA patients. We advise clinicians and researchers to use wearable accelerometers as no physical activity questionnaire has proven to be valid and reproducible in this population.

## Competing interests

The authors declare that they have no competing interests.

## Authors’ contributions

SB, NCC, FMI, NAM gave substantial contribution to the study conception and design. SB performed data acquisition, and all authors performed data analysis and interpretation. SB drafted the article, and all the authors revised it critically for important intellectual content and gave final approval.

## Pre-publication history

The pre-publication history for this paper can be accessed here:

http://www.biomedcentral.com/1471-2474/15/46/prepub
